# Assessing farmers’ perspective on antibiotic usage and management practices in small-scale layer farms of Mymensingh district, Bangladesh

**DOI:** 10.14202/vetworld.2019.1441-1447

**Published:** 2019-09

**Authors:** Jannatul Ferdous, Sabbya Sachi, Zakaria Al Noman, S. M. Azizul Karim Hussani, Yousuf Ali Sarker, Mahmudul Hasan Sikder

**Affiliations:** Department of Pharmacology, Faculty of Veterinary Science, Bangladesh Agricultural University, Mymensingh - 2202, Bangladesh

**Keywords:** antibiotic usage, AWaRe categorization, layer farms, management practices

## Abstract

**Background and Aim:**

Indiscriminate and injudicious use of antibiotics in layer farms is a common practice of Bangladesh for the compensation of Poor management practices and ignorance. Despite this scenario, there is no published documentation on antibiotic usage pattern and farm management practices in layer farms. This study was undertaken to understand the farmers’ perspective in small-scale layer farms regarding antibiotics usage and farm management.

**Materials and Methods:**

A questionnaire survey was conducted in 120 small-scale layer farms of Mymensingh district during January-February 2017. We only considered farms in production. Data were analyzed on antibiotic usage, purpose, egg management, understanding of antibiotic residue, withdrawal period, and other issues.

**Results:**

Among 120 farmers, about 94.16% of farmers are using antibiotics without respecting the withdrawal period. Only 39.1% of farmers possess knowledge of residues. In our surveyed farms, 91.83% of farmers are not practicing egg washing before supplying to the market and 52.67% of farmers are unaware of cleaning and disinfection of egg tray. Ten different types of antibiotics of seven classes have found in the survey. Most antibiotics are in the Watch (49%) and Reserve (8%) groups according to the WHO AWaRe categorization and 73% antibiotics are critically important for human medicine and are considered as last resort.

**Conclusion:**

This study found that due to the lack of knowledge and poor management, farmers consider using antibiotics as the most effective practices to control disease and enhancement of egg production. These indiscriminate uses of antibiotics are responsible for antibiotic residual and resistance problem. Here, we also provide some suggestion and guidelines to improve management practices to minimize the emerging problems of antimicrobial resistance through small-scale layer farms.

## Introduction

Commercial poultry production in Bangladesh is growing rapidly since 1990. There are about 0.15 million commercial poultry farms (broiler and layer) in Bangladesh [1] and at least 50% of those are layer farms [2]. Antibiotics are commonly used at a subtherapeutic level in the poultry industry due to its growth-promoting effect. Delivered in the feed or water, these antibiotics stabilize the gut population and reduce susceptibility to disease [3]. Laying hens may be exposed to veterinary drugs due to (a) therapeutic use after proper diagnosis, (b) control and prevention of disease, (c) growth promotion, (d) stimulation of egg production, (e) illegal or extra-label use of drugs, (f) cross-contaminated during feed mixing unintentionally, and (g) the use of mislabeled feed [4-7]. Commonly used antibiotics in veterinary practices are aminoglycosides, tetracyclines, beta-lactams, quinolones, macrolides, polypeptides, amphenicols, and sulfa drugs [8]. Eggs produced by laying hens which have been given antibiotics can have residual levels of these compounds in the egg yolk and albumen [9].

The egg is considered one of the most nutritious foods which are cheap and readily available [10]. The presence of antibiotic residue in the egg may lead to pathogen resistance to antibiotics used in human medicine. [11]. Nowadays, the main concern of the sanitary authority is to protect the public health against possible harmful effects of these veterinary drugs [12]. Modern animal production practices in Bangladesh and many countries are associated with regular use of antimicrobials, potentially increasing selection pressure on bacteria to become resistant. Globally, average estimated annual consumption of antimicrobials per kilogram in poultry is 148 mg/kg [13]. According to the Bangladesh Veterinary Practitioners Ordinance, 1982, only registered veterinarians are allowed to prescribe medicine or to perform surgery. However, not only registered veterinarian but also non-veterinarian personnel and farmers used antimicrobials without a confirmatory diagnosis or for growth promotion purposes [14]. According to the Drug Act 1940, only registered pharmacists are allowed to sell antibiotics with the documentation of legal prescription. In Bangladesh, so far, there are no specific guidelines for using antibiotic in laying hens. Antibiotics are indiscriminately used as growth promoters and to control infectious diseases in poultry [15]. These unjustified usages are considered as the most vital selecting force to antimicrobial resistance (AMR) in bacteria. Due to the enormous use of antibiotics in the field of poultry, an increased number of resistant bacterial strains were developed in recent years [16]. However, there is a little known about the farmer’s perceptions on indiscriminate use of antibiotics in layer industry and AMR. National sales data are unavailable in Bangladesh though it is the first step to analyze the types of antibiotics or species of animal indicated. However, sales data do not explain about the frequency of antibiotic use, route of administration, farmer’s knowledge of prescription pattern, antibiotic storage facilities, antibiotic residue, withdrawal period, shelf-life of antibiotics, egg management, etc.

The study was designed to learn what is known to the small-scale poultry farmers of Mymensingh district, Bangladesh, and their perception regarding antibiotic usage. Proper understanding of these factors leads to indiscriminate use of antibiotics and antibiotics resistance which, in turn, can minimize AMR. This is the first report in Bangladesh where we collected and analyzed data regarding veterinary antibiotic usage in layer farms.

## Materials and Methods

### Ethical approval

Ethical approval is not required to pursue this study.

### Informed consent

Selected poultry farmers were approached properly and explained about the research before the study was conducted. Informed consent was obtained from all the participants of this study.

### Study area

**Figure-1:**
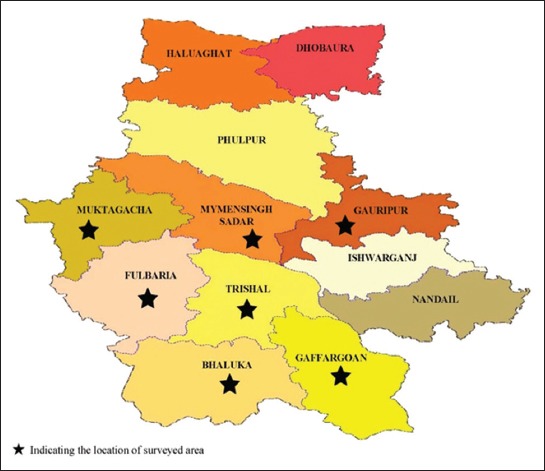
Location of surveyed layer farms in Mymensingh district, Bangladesh [17].

The study was conducted in Mymensingh district, covering seven Upazilas (administrative regions), namely, Fulbaria, Muktagacha, Gauripur, Gaffargaon, Mymensingh Sadar, Trishal, and Bhaluka. Location of each Upazilas is presented in Figure-1 [17].

### Selection of farms

A total of 120 small-scale layer farms were selected randomly for the survey, which were in production. Poultry shed covering 500-1500 ft in size was grouped as small-scale farm [18]. We have surveyed 14 farms from Fulbaria, 17 farms from Muktagacha, 13 farms from Gauripur, 13 from Gaffargaon, 16 farms from Mymensingh Sadar, 21 farms from Trishal, and 26 farms from Bhaluka.

### Preparation of questionnaire

A semi-structured questionnaire was designed to collect relevant information to assess farmers’ knowledge and management procedures in farms related to antibiotic usage and residual problem in the egg that may lead to residue in human food chain. The questionnaire was written both in English and Bangla. All questions in the questionnaire were pre-tested to test the suitability of this script to the target group before finalization.

### Collection of data

All necessary information was collected through personal interview of the farm owner or manager in Bangla on the basis of the questionnaire. Data were collected in January-February 2017. Data were collected on the following criteria: (i) knowledge regarding antibiotic residue; (ii) duration and withdrawal period of antimicrobials; (iii) types of antimicrobial used; (iv) purpose of antibiotic use; (v) egg management; (vi) route of administration of antibiotics; (vii) obtaining suggestions regarding antibiotics usage, etc. Some additional information was collected on total population in farms, age of birds, number of sheds, educational status of the respondent, etc. During the survey, all the leftover packets and bottles of antibiotics were collected and recorded for obtaining accurate information regarding antibiotic usage. Each interview was double-checked to identify any laps and gaps of information and was corrected whenever necessary.

### Statistical analysis

Surveyed data were stored in Microsoft Excel 2010 and further analysis was done by SPSS IBM 20 for descriptive statistics (IBM Corp. Released 2011, IBM SPSS Statistics for Windows, Version 20, Armonk, New York, USA: IBM Corp). Responses to the questionnaire are presented in simple frequency. Graphs were prepared by GraphPad Prism 6 (GraphPad Software, La Jolla, California, USA, www.graphpad.com).

## Results

A total of 120 small-scale layer farms were surveyed for the study with a semi-structured questionnaire. Around 30 farms were not included either these were not in production or farmers refused to participate. All the data were collected and analyzed to assess the farmers perspective on antibiotics usages and factors related to AMR.

### Farmer’s educational status, knowledge of antibiotic residue, shelf-life, aware of the withdrawal period, and storage of drug

Educational statuses of selected farmers were primary to graduate; most had education up to secondary (56%). A good number of farmers have knowledge of antibiotic residue (73%) and shelf-life (96%); however, few are aware of the withdrawal period (7%) and proper storage of drugs (around 40%). Data are summarized in Table-1.

**Table 1:** Farmer’s educational status, knowledge of antibiotic residue and shelf-life, aware of withdrawal period, and storage of drug (n=120).

Educational status		Knowledge of antibiotic residue		Knowledge of shelf-life,		Aware of withdrawal period		Storage of drug	
				
Level of education	Number	Yes	No	Yes	No	Yes	No	Place	Number
Illiterate	5 (4)							Storage room	23 (19.17)
Primary	16 (14)								
Junior secondary	12 (10)	73 (60.83)	47 (39.1)	96 (80)	24 (20)	7 (5.83)	113 (94.16)	Poultry shed	60 (50)
Secondary	65 (54)								
Higher secondary /diploma/	6 (5)							Refrigerator	37 (30.83)
Graduate	16 (13)								

### Egg management, vaccination schedule, and monitoring of antibiotic residues

Farmers were asked about vaccination, egg management including washing of eggs (wet cleaning) [19] and egg trays management and regarding monitoring of antibiotic residues. All farmers were maintaining the vaccination schedules. In respect to egg management, only 4.16% among 120 farmers were washing eggs before supply to wholesaler or other places. Regarding egg tray management, we asked about cleaning and disinfection of egg tray. Only 0.83% of farmers were practicing cleaning and disinfection regularly, 47.5% of farmers sometimes did, and 51.67% of farmers were not doing any management of the egg tray. We assessed farmer’s knowledge of antibiotic residue. No authorities are monitoring residue of antibiotics in egg. Even no one from the Department of Livestock Services or NGO is raising awareness of residue and its effect. Most of the farmers are not aware of antibiotic residue.

### Types of antibiotics use

**Figure-2:**
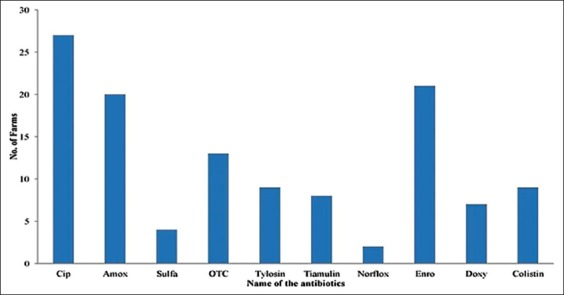
Frequency of antibiotics used in the surveyed layer farms.

Ten different types of antibiotic were found among the 120 poultry farms. Most of the farms are multidrug users. Our survey found that ciprofloxacin, amoxicillin, sulfa drugs, oxytetracycline, tylosin tartrate, tiamulin, norfloxacin, enrofloxacin, doxycycline, and colistin sulfate are being used on the farm. The percentages of these antibiotics usage pattern are shown in Figure-2.

**Figure-3:**
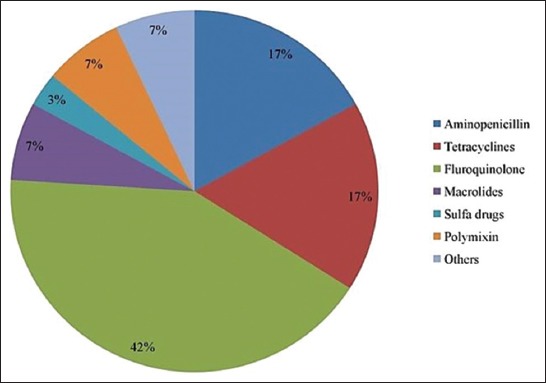
Percentage of different class of antibiotics in the surveyed layer farms.

**Figure-4:**
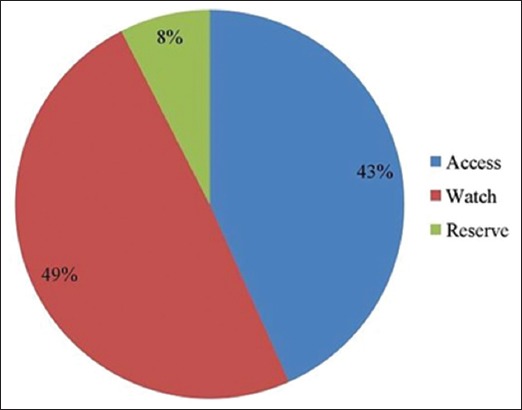
Classification of antibiotics on the basis of the AWaRe categorization of antibiotics.

**Figure-5:**
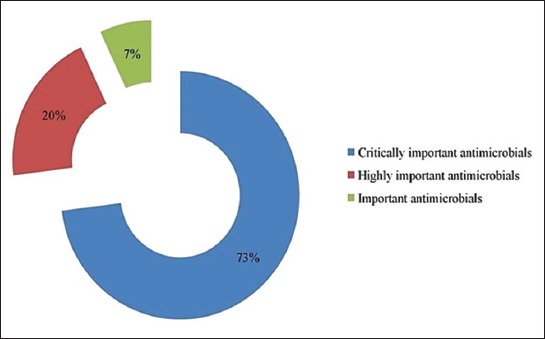
Classification of the antimicrobials used by surveyed poultry farms according to the FAO, WHO, and OIE criteria for critically important antimicrobials for human medicine.

The most common antibiotics in surveyed farms were ciprofloxacin (22.5%), followed by enrofloxacin (17.5%), amoxicillin (16.66%), oxytetracycline (10.83%), sulfa drugs (3.33%), and norfloxacin (1.66%). Class-wise most antibiotics belong to fluoroquinolones, followed by tetracyclines, aminopenicillin, and polymyxin and rest are in macrolides and sulfa groups (Figure-3). Among them, fluoroquinolones, aminopenicillin, and sulfa drugs are not FDA-approved antibiotic for laying hens in many countries due to lessen the prevalence of resistant bacteria [20]. We have further classified the antibiotics usage pattern into Access, Watch, and Reserve antibiotic according to the WHO essential medicine list [21] (Figure-4). Then, we have categorized the antibiotics as critically important, highly important, and important antimicrobials group (Figure-5).

### Purpose, frequency, route, course completion, and idea of using antibiotics

Farmers used antibiotics for both therapeutics and prophylaxis purposes (Table-2) and mostly when needed (80%) through drinking water (75.83%). Most farmers (92.05%) complete the course of antibiotics. Some farmers (18.34%) indicated that they administered antibiotics to their own poultry based on experience or copying from an old prescription.

**Table 2:** Purpose of the use of antibiotics, frequency of use, route of administration, course completion, and idea of using antibiotics.

Purpose of antibiotics use		Frequency of use		Route of administration		Course completion		Idea of using antibiotics	
				
Purpose	Number	Parameter	Number	Parameter	Number	Yes	No	Parameter	Number
Egg production fall	13 (10.83)								
Therapeutics	41 (34.16)	Daily	24 (20)	Water	91 (75.83)	111 (92.5)	9 (7.5)	Prescribed	98 (81.66)
Prophylaxis	17 (14.17)	When needed	96 (80)	Feed and water	29 (24.16)			Self	22 (18.34)
Both (therapeutic and prophylaxis)	49 (40.83)								

## Discussion

We studied farmers’ perspective on antibiotic usages in small-scale layer farm in Mymensingh district of Bangladesh. Our sample size was comparatively small as we have only considered layer farm in production at the time of the survey. In a few cases, farmers were uncooperative to the surveyor and were not included in the survey.

Most of the small-scale layer farmers are educated though only a few take necessary steps to minimize residue. About 80% of farmers are aware of shelf-life of drugs as they want the best action of antibiotics for their layer birds, but almost all the farmers do not respect the withdrawal period. Farmers complain that no veterinarian provides them the knowledge of the withdrawal period or some farmers think that government veterinarians are not cooperative and easily assessable [22]. Although veterinarians are aware of withdrawal period and residual knowledge, they do not create mass awareness due to socioeconomic constraints of the country as farmers have no insurance or governmental support for the loss of their business. Even those (5.83% of farmers) possess knowledge of the withdrawal period; do not follow it during the selling of the eggs. This possesses a potential risk to transfer the drug residue from egg to human body. Furthermore, most farmers did not imply appropriate farm hygiene, biosecurity, and proper antibiotic management to prevent infection or recurrence of disease in the farm [14,23].

The farmers of Mymensingh district are not aware of the quality management of eggs and egg tray through cleaning and disinfection before entering into farms as it is a potential source of bacterial entrance. This is not the sign of good management practices in poultry farms [8]. Vaccination does not provide 100% protection against diseases. For the best prevention, strict biosecurity, good housing, and management are essential. As these practices are infirm in farms, so vaccine does not comply with the highest protection. In addition, vaccination schedules often did not match with local pattern of disease, level of biosecurity, and level of challenge in each sector of operation of production [24].

Almost 100% of farmers in our study are using antibiotic in their farms. This finding is comparable with other studies which reported high usage of antibiotics in poultry production to control the source of infection in farms due to poor management [8,14,25,26]. It was evident that majority of farmers were continuously using antibiotics as therapeutic and prophylaxis and with higher intensity during disease outbreaks. About 20% of farmers use antibiotic daily without any reasons. Most of the farms are prescribed antibiotic by the registered veterinarian and non-veterinarian, but 18.34% of farmers use antibiotic of their own choice and on the basis of experience as antibiotic is easily and unlawfully purchasable from unregistered pharmacy and dealer shop [27]. Non-cooperating behavior of governmental veterinarian and farmers’ ignorance or unavailability of trained veterinarian at the time of disease havoc and immunization push the farmers to prescribe for their own flock [22]. This practice is responsible for indiscriminate use of antibiotics and potential chance to AMR [28]. Indiscriminate use of antibiotic can be alleviated by increasing veterinarian responsibility, training program, and mass awareness and monitoring. The quality of antibiotics differs from one pharmaceutical to another. Poor grade antibiotics are unable to kill whole bacterial colony. This leads potential risk to AMR. Dishonest practitioners influenced by persuasive marketing policy of pharmaceuticals sometimes prescribe poor quality antibiotics. Moreover few farmers prescribe antibiotics for their flock instead of veterinarian’s suggestion could lead to selection of poor quality antibiotics [29]. The bigger picture lies in lack of public health education, awareness program for food safety, and enforcement of laws and legislation.

Multidrug using is common in poultry farms with the practice of broad-spectrum antibiotics. Ciprofloxacin and norfloxacin were the highest and lowest used drug, respectively. Fluoroquinolones are the top-ranked antibiotic classes followed by tetracyclines, aminopenicillin, and polymyxin groups, suggesting similar observations from other studies [14,26,30]. In Bangladesh, the prevalent bacterial diseases of poultry are salmonellosis, colibacillosis, fowl cholera, and mycoplasmosis [31]. However, selection of antibiotics and disease prevalence does not match with each other due to choosing broad-spectrum antibiotics without proper justification or choosing of antibiotics which are available in powder form that is easily administrable through drinking water. This phenomenon is also similar globally. We found that 75.83% of farmers are practicing mixing antibiotics into drinking water. Rest are using feed and water to mix antibiotic at the same time. No farmers depend on feed to administer antibiotics as it causes uneven distribution of antibiotics. Furthermore, sick birds are more likely to continue to drink water rather than have feed [8] and farmers want the laying birds to complete the course.

Our surveyed farms are using most of antibiotic from Watch groups including fluoroquinolones and macrolides. These antibiotics have higher resistance potentials and critically important antimicrobials for human medicine (CIA list) and apply for risk management strategies for the use of antimicrobials in food production animals [32]. Furthermore, 8% of used antibiotics are in Reserve groups which are considered as “last resort” for multidrug-resistant (MDR) treatment in human and generally not recommended for livestock and poultry. By exposing these two groups, we are creating a potential space to make superbug and MDR bacteria.

Our questionnaire survey response does not, therefore, cover the entire antibiotic usage phenomena of the Bangladesh poultry industry; rather, it is only cover the small-scale layer farms. It is very difficult to collect data regarding usage and prescription pattern of antibiotic used in the poultry industry in Bangladesh due to legislation not being practiced here. Data should be collected from more farmers and prescriber’s to assess the bigger picture. However, our study demonstrates the factors and perspective of antibiotic usage pattern in small-scale layer farms in Bangladesh.

## Conclusion

Publicly available, verifiable data on usage of antibiotics in poultry industry and production in Bangladesh are shockingly incomplete. More than 90% of farmers have educational qualification, most of them are aware of shelf-life of drug and compel to course completion but do not respect the withdrawal period. All small-scale layer farms are multidrug users and having prescription from registered veterinarian, non-veterinarian, or even self-medication. Different antibiotics of different classes have observed in those farms including critically important antibiotic and antibiotic considering as last resort from human being which should not be used without definite causes. It is responsible for emergence of antibiotic resistance among the biota. Strict biosecurity, intensive extension, educational program, and governmental legislation on responsible use of antibiotics could lead to restriction on indiscriminate use of antibiotics in layer farms in Bangladesh. Hence, National Residue Control Programs should be implemented. Education and awareness programs have to be started on the use of antibiotics and the risk for AMR across the sector to farmers, veterinarians, sellers of antibiotics, and other stakeholders. These sectors could work together to promote responsible use and promote good practices. National sales data should be collected in regular interval. Legislation of prescribing antibiotics and authorized sales according to guidelines should be enforced. Illegal trade and unauthorized prescribers should be penalized.

## Authors’ Contributions

JF and MHS planned and designed the research work. JF, SS, and ZA carried out the experiments with the collection of data. MHS supervised the whole experiments. SMAKH and YAS helped in drafting and revised the manuscripts for important intellectual analysis. All authors read and approved the final manuscript.
